# Combinatorial IL-17RB, ST2, and TSLPR Signaling in Dendritic Cells of Patients With Allergic Rhinitis

**DOI:** 10.3389/fcell.2020.00207

**Published:** 2020-04-03

**Authors:** Rui Zheng, Yang Chen, Jianbo Shi, Kai Wang, Xuekun Huang, Yueqi Sun, Qintai Yang

**Affiliations:** ^1^Department of Otorhinolaryngology-Head and Neck Surgery, The Third Affiliated Hospital, Sun Yat-sen University, Guangzhou, China; ^2^Otorhinolaryngology Hospital, The First Affiliated Hospital, Sun Yat-sen University, Guangzhou, China; ^3^Department of Otorhinolaryngology-Head and Neck Surgery, First People’s Hospital of Foshan, Foshan, China; ^4^Department of Otolaryngology, The Seventh Affiliated Hospital, Sun Yat-sen University, Shenzhen, China

**Keywords:** interleukin-17 receptor B, interleukin-33 receptor, thymic stromal lymphopoietin receptor, dendritic cells, allergic rhinitis

## Abstract

**Objectives:**

Myeloid dendritic cells (DCs) in patients with allergic rhinitis (AR) express higher levels of IL-17RB, ST2, and TSLPR. However, their functional roles in DCs are much less clear. This study aimed to determine the combined effects of these three receptor signals on the T cell-polarizing function of DCs in AR patients.

**Methods:**

Monocyte-derived DCs (mo-DCs) were generated and stimulated with Toll-like receptor (TLR) 1–9 ligands. Der.p1-induced mo-DCs were stimulated with different combinations of IL-25, IL-33, and TSLP to determine phenotypic characteristics and then co-cultured with CD4^+^ T cells to assess Th2 cytokine production. Expression levels of IL-17RB, ST2, and TSLPR on myeloid DCs (mDCs) from peripheral blood of AR and healthy subjects were detected to confirm the association of these receptors with disease severity.

**Results:**

TLR ligands induced AR-derived mo-DCs to increase IL-17RB, ST2, and TSLPR expression by varying degrees; among these, Der.p1 was the strongest inducer. Der.p1-induced mo-DCs from AR showed increased OX40L expression. IL-25, IL-33, and TSLP (alone or in double combination) significantly increased OX40L expression on Der.p1-induced mo-DCs from AR, thereby increasing the production of IL-4, IL-5, and IL-13 in co-cultured CD4^+^ T cells; triple combination further enhanced these effects. The percentage of IL-17RB^+^ST2^+^TSLPR^+^ mDCs was increased in AR, higher in moderate to severe phase than in mild phase, and positively correlated with the percentages of IL-4^+^, IL-5^+^, and IL-13^+^ T cells.

**Conclusion:**

A combination of IL-17RB, ST2, and TSLPR signals amplified the Th2-polarizing function of DCs and was associated with disease severity in AR patients.

## Introduction

Allergic rhinitis (AR) is a prevalent disease both in China and Western countries, affecting approximately 10–40% of people worldwide (Salo et al., 2014; Zuberbier et al., 2014; Zhang and Zhang, 2019). Pathophysiologically, AR is an IgE-mediated type 2 inflammatory disorder caused by the interaction of airborne allergens. AR is characterized by inflammatory infiltrates, predominantly comprising eosinophils, mast cells, basophils, and T cells, which release granule proteins, cytokines, and chemokines to trigger the onset of clinical symptoms such as rhinorrhea, sneezing, nasal itching, and nasal congestion (Bousquet et al., 2008; Cheng et al., 2018; Wise et al., 2018). AR severity is typically classified as either mild (M-AR) or moderate to severe (MS-AR) based on symptom severity according to Allergic Rhinitis and its Impact on Asthma (ARIA) guidelines (Bousquet et al., 2008). Over half of AR patients are MS-AR, leading to significantly impaired normal daily activity (Canonica et al., 2007; Schatz, 2007). However, the mechanism driving the development of this form remains unclear.

Dendritic cells (DCs) are the most potent antigen-presenting cells; they play a pivotal role in the initiation of adaptive T cell immunity (Rossi and Young, 2005). In particular, myeloid DCs (mDCs), identified by the specific surface marker CD1c, are a major subset of DCs that preferentially activate and induce polarization of CD4^+^ T cells. Studies have indicated that mDCs are essential for the priming of Th2 cells, which are linked to anti-parasite immunity and are also associated with inappropriate response to innocuous antigens, triggering an allergic immune response (Lambrecht et al., 2000; Phythian-Adams et al., 2010; Froidure et al., 2014; Hussaarts et al., 2014). Accumulating evidence has revealed that signals from DCs play an essential role in Th2-cell polarization in allergic airway diseases (Froidure et al., 2014; Hussaarts et al., 2014; Tjota and Sperling, 2014). IL-25, IL-33, and thymic stromal lymphopoietin (TSLP) are three innate cytokines that are mainly derived from epithelial cells. Previous studies have shown that this triad of cytokines plays a role in Th2 cell maturation through DC activation, thereby enhancing their Th2-polarizing capacity. For example, TSLP was able to activate human nasal mucosal CD1^+^ DCs and enhance their capacity to initiate Th2 responses (Melum et al., 2014). In a mouse model of ovalbumin-induced asthma, IL-25 was reported to promote DC activation, inducing naïve T cells to differentiate into pro-inflammatory Th2 cells (Hongjia et al., 2014). Upon exposure to IL-33, DCs exhibited increased protein expression of CD40 and OX40 ligand (OX40L), thereby potently inducing Th2 responses (Besnard et al., 2011). Although these studies have demonstrated that IL-25, IL-33, and TSLP can independently develop type 2 immunity, more recent studies have shown that combined targeting of this cytokine triad is necessary for the suppression of progressive Th2-driven inflammation (Van Dyken et al., 2016; Vannella et al., 2016; Khodoun et al., 2018), suggesting that the functional activities of these three cytokines do not overlap.

Monocyte-derived DCs (mo-DCs), which can be generated from peripheral blood monocytes *in vitro* by 5–7 days of culture with GM-CSF and IL-4, have been regarded as an important source of inflammatory DCs during pathologic inflammation and closely simulate mDCs found in airway mucosa (Huang et al., 2011; Hu et al., 2013), thus providing a potent tool for the study of the role of mDC in allergic inflammation. Recently, we reported using this cell model that CD1c^+^ mo-DCs derived from AR patients expressed higher levels of IL-17RB (IL-25 receptor), ST2 (IL-33 receptor), and TSLPR (TSLP receptor) than healthy control (HC) subjects (Zheng et al., 2017). However, the relative contribution and importance of each of these cytokines in directing the Th-polarizing function of DCs have yet to be conclusively established. The aim of this study was to determine the combined effect of IL-25, IL-33, and TSLP on DC function.

## Materials and Methods

### Subjects

Volunteers with AR and healthy volunteers with no allergic or autoimmune disease symptoms were enrolled at Otorhinolaryngology Hospital, The First Affiliated Hospital, Sun Yat-sen University, China, from November 2017 to March 2019. AR diagnosis was made according to ARIA guidelines. Atopic status was evaluated by the concentrations of serum IgE (ImmunoCAP, Phadia, Uppsala, Sweden) specific to local common inhalant allergens; e.g., house dust mites (HDMs), pets, pollens, molds, cockroaches, and so on (Johansson, 2004). Concentrations above 0.7 IU/mL were considered positive. AR subjects mono-sensitized to HDMs and HC subjects negative to all allergens were screened. Subjects who received oral glucocorticoids, antihistamines, or immunotherapy, or had experienced acute upper and lower respiratory tract infection during the past month were excluded. A total of 15 AR and 10 HC subjects were included in this study; their characteristics are listed in Table 1. Peripheral blood samples for *in vitro* cell culture studies were collected from 10 AR and 10 HC subjects. Due to the limited amount of blood samples, not all samples were included in every study protocol. The 15 AR patients all underwent long-term follow-up as described below. This study was approved by the Ethics Committee of the First Affiliated Hospital, Sun Yat-sen University. All subjects provided written informed consent before participation. Standard biosecurity and institutional safety procedures were adhered to throughout the study.

**TABLE 1:** Clinical characteristics of HC and AR subjects.

	**HC subjects**	**AR subjects**		***P*-value**		

Number	10	15		N/A		
Age (y), median (range)	25 (22–27)	25 (20–31)		0.819		
Gender (male/female)	4/6	7/8		N/A		

		**M-AR**	**MS-AR**	**HC vs. M-AR**	**HC vs. MS-AR**	**M-AR vs. MS-AR**
HDM-specific IgE (IU/mL), mean (SD)	0.14 (0.09)	28.42 (22.69)	54.82 (38.32)	0.024	0.013	0.036
Blood EOS absolute number (× 10^9^/L), mean (SD)	0.11 (0.06)	0.21 (0.11)	0.34 (0.23)	0.042	0.007	0.043
TNSS, median (IQR)	N/A	3 (1)	7 (2)	N/A	N/A	<0.001

*HC, healthy control; AR, allergic rhinitis; M-AR, mild AR; MS-AR, moderate to severe AR; HDM, house dust mite; EOS, eosinophil; TNSS, Total Nasal Symptom Score; SD, standard deviation; IQR, interquartile ranges; N/A, not applicable.*

### Follow-Up Study Design

Screening phase. At the time of screening (visit 1), all AR patients were asked to complete a Total Nasal Symptom Score (TNSS) questionnaire to assess disease severity.

Mild phase. AR was classified as M-AR when TNSS ≤ 4 and MS-AR when TNSS ≥ 5. Subjects were instructed to contact the study team if they experienced persistent M-AR for 2 weeks (visit 2).

Moderate to severe phase. A final visit (visit 3) occurred 2 weeks after transition into MS-AR.

Peripheral blood samples for specific IgE detection, blood routine testing, flow cytometry, and enzyme-linked immunosorbent assay (ELISA) were collected on visits 2 and 3.

### TNSS

The TNSS was completed by subjects based on a 1-week recall. Subjects were asked to assign scores that best reflected the intensity of nasal itching, sneezing, rhinorrhea, and nasal congestion caused by AR. Scoring criteria were as follows: 0, no symptoms; 1, occasional mild symptoms; 2, frequent moderate symptoms; and 3, continuous severe symptoms. The TNSS was calculated (range: 0–12) by summing the individual nasal scores.

### Generation and Culture of DCs

To investigate the association of IL-17RB, ST2, and TSLPR on mDC with AR severity, peripheral blood mononuclear cells (PBMCs) were isolated for flow cytometry from whole-blood samples of HC, M-AR, and MS-AR subjects using the Ficoll-Hypaque gradient centrifugation technique, with erythrocytes removed using RBC Lysis Buffer (eBioscience, San Diego, CA, United States). CD1c^+^ cells were defined as mDCs.

Generation and culture of mo-DCs were performed as previously described (Hu et al., 2013). Briefly, CD14^+^ monocytes were positively sorted from PBMCs using the immunomagnetic cell sorting method with anti-CD14 antibody-coated magnetic beads (Miltenyi Biotec, Auburn, CA, United States). CD14^+^ cell purity was > 95%, as confirmed by flow cytometry. To generate DCs, purified monocytes were cultured at a concentration of 5 × 10^5^ cells/mL for 5 days at 37°C in humidified air containing 5% CO_2_, in RPMI 1640 medium supplemented with heat-inactivated fetal bovine serum (FBS) (10%) and penicillin/streptomycin (1%) (all from Gibco, Carlsbad, CA, United States), in the presence of 50 ng/mL recombinant human (rh) granulocyte-macrophage colony-stimulating factor (GM-CSF; PeproTech Inc., Rocky Hill, NJ, United States) and 10 ng/mL rh IL-4 (R&D Systems, Minneapolis, MN, United States). Half of the medium was replaced every 2 days. CD14^–^CD1a^+^ cells were defined as DCs.

To investigate the effects of Toll-like receptor (TLR) ligands on DCs, mo-DCs were cultured for 48 h for flow cytometry in the presence of 300 ng/mL Pam3CSK4, 1 μg/mL PGN-BS, 10 μg/mL Poly I:C, 100 ng/mL FLA-ST, 100 ng/mL FSL-1, 2 μg/mL Imiquimod-R837, 500 ng/mL ssRNA40/LyoVecTM, and 7 μg/mL ODN2395 (all from InvivoGen, San Diego, CA, United States), or 1 μg/mL natural Dermatophagoides pteronyssinus allergen 1 (Der.p1) (Indoor Biotechnologies, Charlottesville, VA, United States). To investigate the combined effects of epithelial cell-derived cytokines on Der.p1-activated DCs, Der.p1-stimulated mo-DCs were further stimulated for 24 h for flow cytometry with different combinations of 10 ng/mL rhIL-25 (catalog no. 1258-IL), 10 ng/mL rhIL-33 (catalog no. 3625-IL), and 100 ng/mL TSLP (catalog no. 1398-TS) (all from R&D Systems).

### Co-culture of DCs With CD4^+^ T Cells

To investigate the combined effects of epithelial cell-derived cytokines on the T cell-polarizing function of DCs, on day 5, mo-DCs were stimulated for 48 h with 1 μg/mL Der.p1 (Indoor Biotechnologies), simultaneously with different combinations of blocking antibodies, namely, 2 μg/mL anti-IL-17RB (catalog no. MAB1207), 1 μg/mL anti-ST2 (catalog no. MAB523), and 1 μg/mL anti-TSLPR (catalog no. AF981) monoclonal antibodies (all from R&D Systems). Then, the mo-DCs were stimulated for an additional 24 h with different combinations of rhIL-25 (10 ng/mL), rhIL-33 (10 ng/mL), and rhTSLP (100 ng/mL) (all from R&D Systems), prior to co-culture with CD4^+^ T cells. On day 8, heterologous CD4^+^ T cells from PBMCs of HC subjects were positively purified by immunomagnetic cell sorting with anti-CD4 antibody-coated magnetic beads (Miltenyi Biotec) as previously described. The purity was > 95%, as confirmed by flow cytometry. The CD69 expression on CD4^+^ T cells in PBMCs, as well as on freshly sorted CD4^+^ T cells, was confirmed by flow cytometry. Stimulated mo-DCs were washed twice with phosphate-buffered saline (PBS) and then co-cultured (5 × 10^4^ per well) with CD4^+^ T cells at a 1:10 DC/T-cell ratio in RPMI 1640 medium supplemented with FBS (10%) and penicillin/streptomycin (1%) (all from Gibco). After 5 days, cells and supernatants were harvested for flow cytometry and ELISA, respectively. For intracellular staining, co-cultured cells were re-stimulated with Cell Stimulation Cocktail (plus protein transport inhibitors) (eBioscience) for the final 5 h; the cocktail consisted of 81 nM phorbol 12-myristate 13-acetate and 1.34 μM ionomycin to stimulate cells to produce cytokines, as well as 10.6 μM brefeldin A and 2 μM monensin to block cytokines in the cytoplasm and enhance intracellular staining signals.

### Flow Cytometry

Cells were suspended in PBS with 0.5% bovine serum albumin (MP Biomedicals, Palm Springs, CA, United States) to prevent unspecific binding, and stained with a combination of florescence-conjugated antibodies. Prior to intracellular cytokine staining, cells were fixed and permeabilized with the Intracellular Fixation and Permeabilization Buffer Set (eBioscience). Detailed information on antibodies used in this study is provided in Table 2. Equal concentrations of species- and subtype-matched antibodies were used as negative controls. Flow cytometry was performed using a Gallios flow cytometer (Beckman Coulter), and data were analyzed using Kaluza Analysis v. 2.1 software (Kaluza Software, Fullerton, CA, United States).

**TABLE 2:** Antibodies used for flow cytometry.

**Antibody**	**Fluorochrome**	**Manufacturer**	**Clone**	**Source**
CD1c	PerCp-Cy5.5	BD Biosciences	F10/21A3	Monoclonal Mouse IgG1, k
CD86	APC	BD Biosciences	FUN-1	Monoclonal Mouse IgG1, k
IL-17RB	PE	R&D Systems	170220	Monoclonal Mouse IgG2b
ST2	APC	R&D Systems	N/A	Polyclonal Goat IgG
TSLPR	Alexa Fluor 700	R&D Systems	147036	Monoclonal Mouse IgG1
OX40L	BV421	BD Biosciences	ik-1	Monoclonal Mouse IgG1, k
PDL1	PE-Cy7	BD Biosciences	MIH1	Monoclonal Mouse IgG1, k
ICOSL	PE	BD Biosciences	2D3/B7-H2	Monoclonal Mouse IgG2b, k
MHC-II	APC	eBioscience	LN3	Monoclonal Mouse IgG2b, k
CD3	PE	BD Biosciences	UCHT1	Monoclonal Mouse IgG1, k
CD4	APC-Cy7	BD Biosciences	RPA-T4	Monoclonal Mouse IgG1, k
IL-4	PerCp-Cy5.5	BD Biosciences	8D4-8	Monoclonal Mouse IgG1, k
IL-5	FITC	R&D Systems	9906	Monoclonal Mouse IgG1
IL-13	BV421	BD Biosciences	JES10-5A2	Monoclonal Rat IgG1
IFN-γ	PE-Cy7	BD Biosciences	B27	Monoclonal Mouse IgG1, k
IL-17A	AF647	BD Biosciences	SCPL1362	Monoclonal Mouse IgG1, k

### ELISA

Cytokine levels in serum and cell culture supernatants were measured using commercial ELISA kits (R&D Systems) according to the manufacturer’s instructions. Lower detection limits are listed in Table 3.

**TABLE 3:** The lower detection limits for ELISA.

**Cytokine**	**Lower detection level (pg/mL)**	**Source**
IL-4	31.2	R&D Systems
IL-5	4.7	R&D Systems
IL-13	93.8	R&D Systems

### Statistical Analyses

PBMC flow cytometry data and serum ELISA data were tested for normality using the Shapiro–Wilk test; data are expressed as means ± standard deviation, or medians with interquartile range according to normality test results. *In vitro* cell culture data are expressed as means ± standard deviation. We used Student’s *t*-test, Mann–Whitney *U-*test, paired *t-*test, or Wilcoxon matched pair signed rank test to assess significant differences among groups. Correlations were analyzed using the Spearman’s rank correlation coefficient. Statistical significance was evaluated at a level of *P* < 0.05.

## Results

### TLR Ligands Induced AR-Derived mo-DCs to Express IL-17RB, ST2, and TSLPR

**FIGURE 1:**
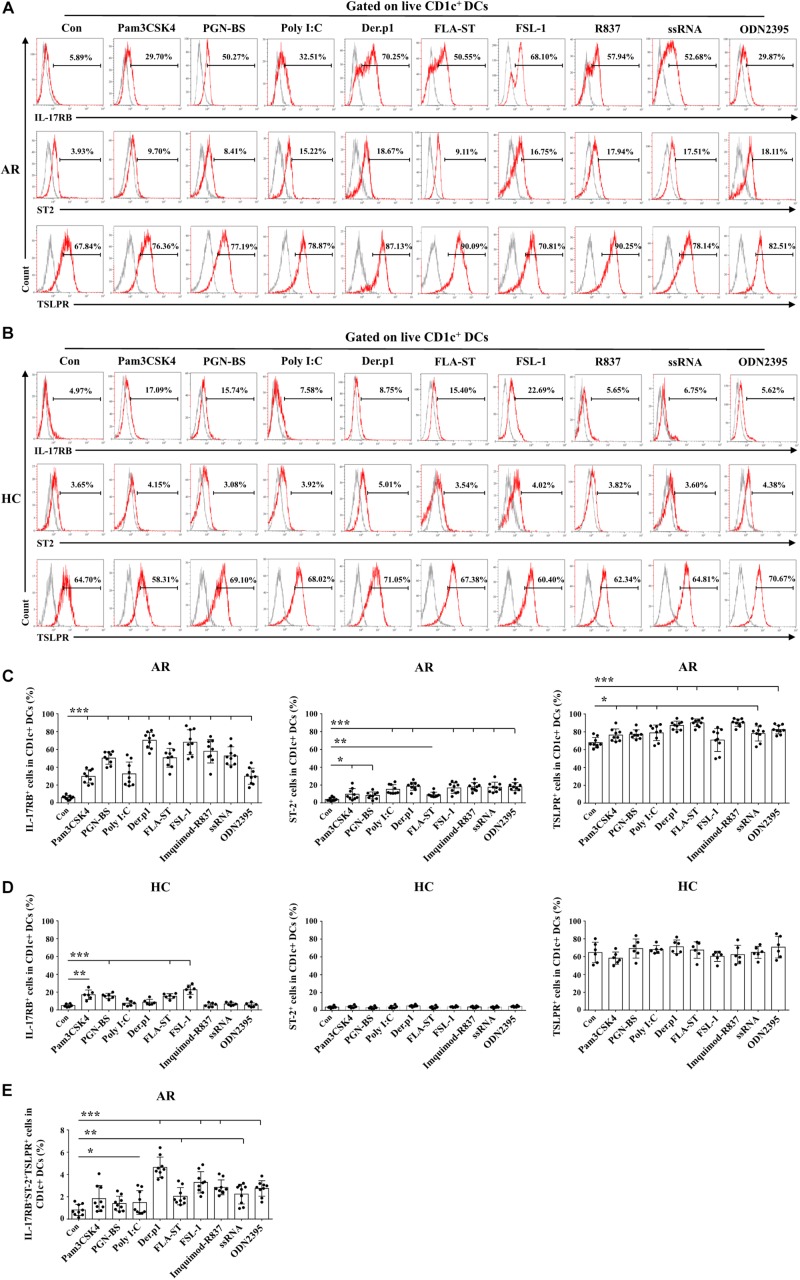
TLR ligands induced allergic rhinitis (AR)-derived monocyte-derived DCs (mo-DCs) to express IL-17RB, ST2, and thymic stromal lymphopoietin (TSLPR). **(A,B)** Representative flow plots of IL-17RB, ST2, and TSLPR expression on CD1c^+^ mo-DCs derived from AR (*n* = 9) and healthy control (HC) (*n* = 6) subjects before and 48 h after stimulation with Pam3CSK4, PGN-BS, Poly I:C, Der.p1, FLA-ST, FSL-1, Imiquimod-R837, ssRNA, and ODN2395. Gray histograms were negative isotypes. **(C,D)** Quantification of percentages of IL-17RB^+^, ST2^+^, and TSLPR^+^ cells in CD1c^+^ mo-DCs derived from AR and HC subjects. **(E)** Quantification of percentages of IL-17RB^+^ST2^+^TSLPR^+^ cells in CD1c^+^ mo-DCs derived from AR subjects. Data are expressed as means ± standard deviation (SD). Significant differences were tested using the paired *t*-test between control and TLR groups, and using Student’s *t*-test between TLR stimulation groups. *P < 0.05, **P < 0.01, ***P < 0.001.

We first extended our recent study (Hu et al., 2013) to explore the effects of TLR ligands other than lipopolysaccharide (LPS) on expression of IL-17RB, ST2, and TSLPR on mo-DCs. In HC-derived mo-DCs, IL-17RB upregulation was observed only with Pam3CSK4, PGN-BS, PLA-ST, and FSL-1 treatment, whereas all TLR ligands tested did not affect ST2 or TSLPR expression (Figures 1B,D). By contrast, all TLR ligands upregulated IL-17RB, ST2, and TSLPR expression on AR-derived mo-DCs by varying degrees; the sole exception was the effect of FSL-1 on TSLPR expression (Figures 1A,C). Der.p1 was the strongest inducer of IL-17RB, ST2, and TLSPR upregulation (Figures 1A,C). The mean percentages of IL-17RB^+^, ST2^+^, and TSLPR^+^ cells in Der.p1-induced mo-DCs were 70.25, 18.67, and 87.13%, respectively (Figure 1A). Der.p1-induced mo-DCs also appeared to express the highest level of co-expression of IL-17RB, ST2, and TSLPR (∼4.64% of Der.p1-induced mo-DCs), compared with the counterparts of other TLR ligand-induced mo-DCs (Figure 1E).

### Phenotypic Characteristics of HDM-Induced mo-DCs

**FIGURE 2:**
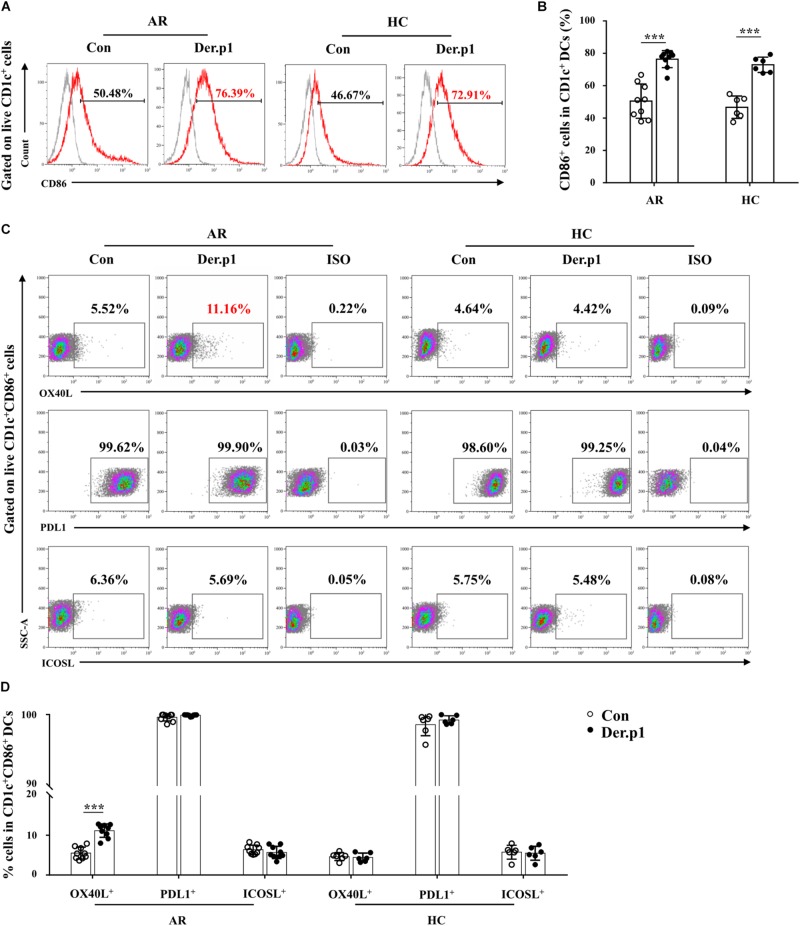
Phenotypic characteristics of house dust mite (HDM)-induced mo-DCs. **(A)** Representative flow plots of CD86 expression on CD1c^+^ mo-DCs derived from AR (*n* = 9) and HC (*n* = 6) subjects before and 48 h after stimulation with Der.p1. Gray histograms were negative isotypes. **(B)** Quantification of percentages of CD86^+^ cells in CD1c^+^ mo-DCs. **(C)** Representative flow plots of OX40L, PDL1, and ICOSL expression on CD1c^+^ mo-DCs derived from AR (*n* = 9) and HC (*n* = 6) subjects before and 48 h after stimulation with Der.p1. **(D)** Quantification of percentages of OX40L^+^, PDL1^+^, and ICOSL^+^ cells in CD1c^+^ mo-DCs. Data are expressed as means ± SD, and tested using the paired *t*-test. ***P < 0.001.

We further characterized the phenotype of Der.p1-induced mo-DCs. We found that Der.p1 upregulated CD86 expression on mo-DCs derived from both HC and AR subjects (Figures 2A,B). However, Der.p1 increased OX40L expression on mo-DCs derived from AR, but not HC subjects (Figures 2C,D). In addition, Der.p1 did not affect the expression of PDL1 or ICOSL on mo-DCs from HC or AR subjects (Figures 2C,D).

### Effects of IL-25, IL-33, and TSLP on the mo-DC Phenotype

**FIGURE 3:**
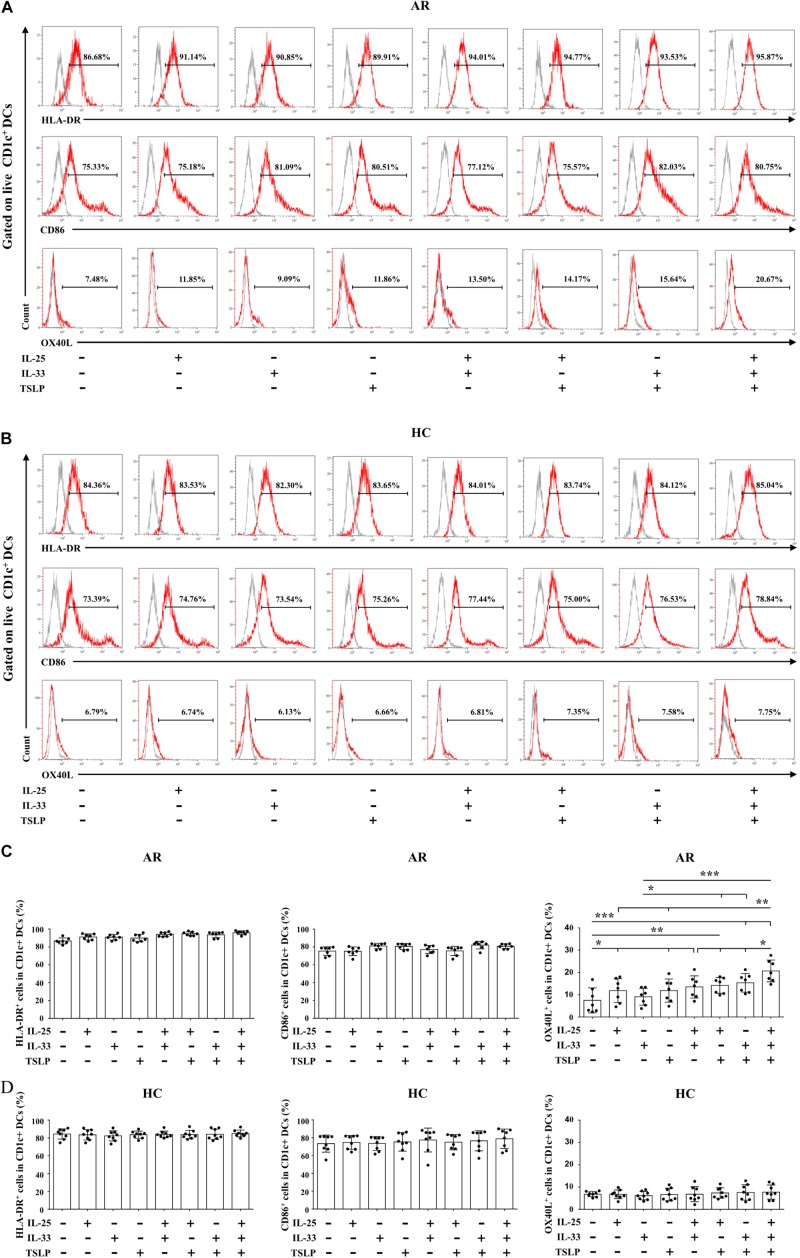
Effects of IL-25, IL-33, and TSLP on the phenotype of mo-DCs. **(A,B)** Representative flow plots of HLA-DR, CD86, and OX40L expression on Der.p1-induced mo-DCs from AR (*n* = 7) and HC (*n* = 8) subjects cultured in the presence of IL-25, IL-33, and TSLP, singly or in combination, for 24 h. Gray histograms were negative isotypes. **(C,D)** Quantification of percentages of HLA-DR^+^, CD86^+^, and OX40L^+^ cells in Der.p1-induced mo-DCs. Data are expressed as means ± SD. Significant differences were tested using the paired *t*-test between control and cytokine stimulation groups, and using Student’s *t*-test between cytokine stimulation groups. *P < 0.05, **P < 0.01, ***P < 0.001.

We next determined the effects of IL-25, IL-33, and TSLP on the phenotype of Der.p1-induced mo-DCs cultured in the presence of IL-25, IL-33, and TSLP, alone or in combination. We observed no significant change in HLA-DR or CD86 expression on mo-DCs from HC or AR subjects cultured with IL-25, IL-33, and TSLP, singly or in combination, compared with those without IL-25, IL-33, and TSLP treatment (Figures 3A–D). Interestingly, increased OX40L expression was observed on AR-derived mo-DCs cultured in the presence of IL-25, IL-33, and TSLP, either alone or in double combination, compared with those cultured in the absence of IL-25, IL-33, and TSLP (Figures 3A,C). Surprisingly, mo-DCs cultured with a triple combination of IL-25, IL-33, and TSLP showed further enhanced OX40L expression (Figures 3A,C). We did not observe similar results on HC-derived mo-DCs (Figures 3B,D).

### Effects of IL-25, IL-33, and TSLP on the Pro-Th2 Function of AR-Derived mo-DCs

**FIGURE 4:**
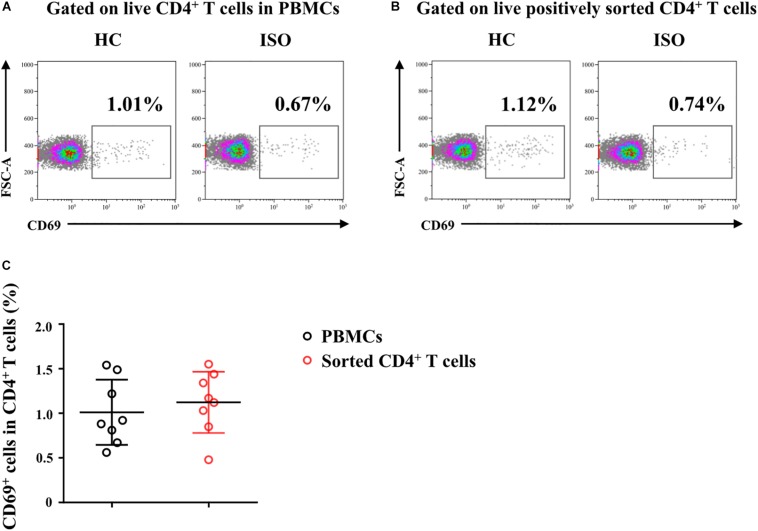
Freshly sorted CD4^+^ T cells from HC subjects were not activated. **(A,B)** Representative flow plots of CD69 expression on CD4^+^ T cells in peripheral blood mononuclear cells (PBMCs) and on freshly positively sorted CD4^+^ T cells from HC subjects (*n* = 8). **(C)** Quantification of percentages of CD69^+^ cells in CD4^+^ T cells in PBMCs and in sorted CD4^+^ T cells. Data were tested for normality using the Shapiro–Wilk test, and expressed as means ± SD according to normality test results. Significant difference was tested using paired *t*-test between groups.

**FIGURE 5:**
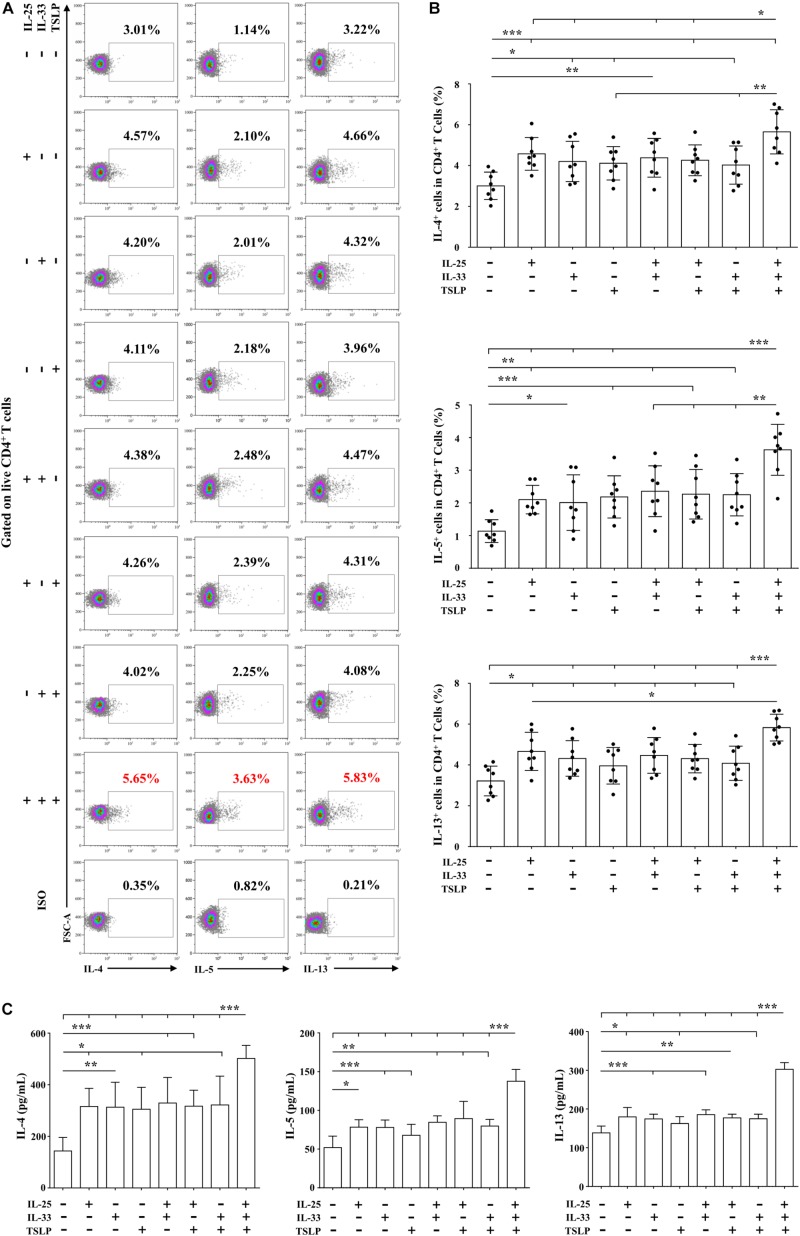
Effects of IL-25, IL-33, and TSLP on the pro-Th2 function of AR-derived mo-DCs. **(A)** Representative flow plots of IL-4, IL-5, and IL-13 expression by CD4^+^ T cells from HC subjects (*n* = 8) co-cultured for 5 days with Der.p1-induced mo-DCs from AR subjects (*n* = 8). Prior to co-culturing, Der.p1-induced mo-DCs were stimulated with IL-25, IL-33, and TSLP, singly or in combination, for 24 h. **(B)** Quantification of percentages of IL-4^+^, IL-5^+^, and IL-13^+^ cells in CD4^+^ T cells. **(C)** Protein levels of IL-4, IL-5, and IL-13 in the supernatants of co-cultured cells as measured by ELISA (*n* = 6). Data are expressed as means ± SD. Significant differences were tested using the paired *t*-test between control and cytokine stimulation groups, and using Student’s *t*-test between cytokine stimulation groups. *P < 0.05, **P < 0.01, ***P < 0.001.

**FIGURE 6:**
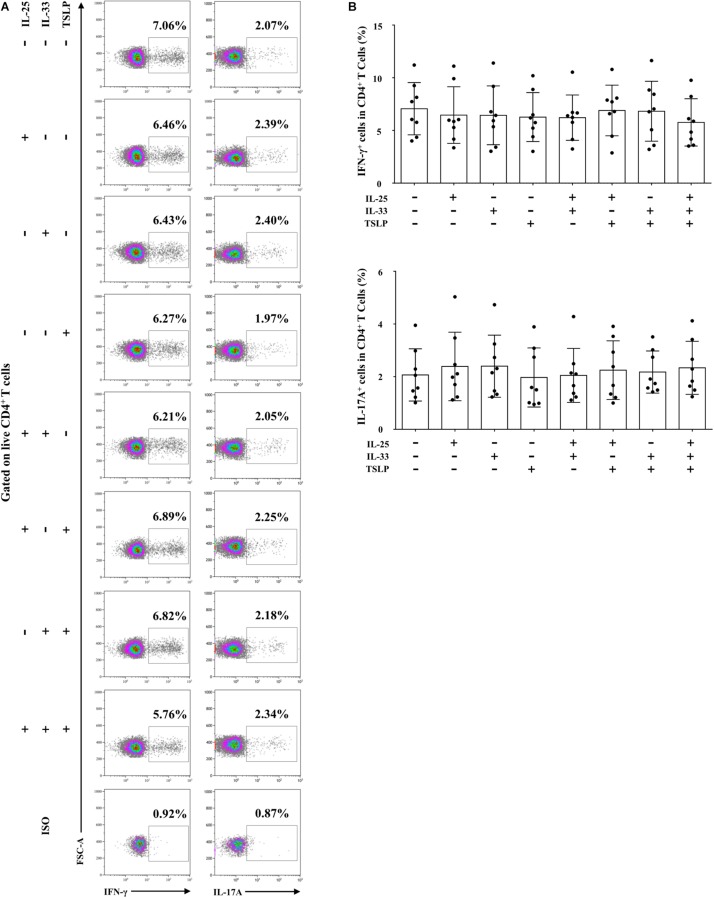
IL-25, IL-33, and TSLP had no effect on the pro-Th1/Th17 function of AR-derived mo-DCs. **(A)** Representative flow plots of IFN-γ and IL-17 expression by CD4^+^ T cells from HC subjects (*n* = 8) co-cultured for 5 days with Der.p1-induced mo-DCs from AR subjects (*n* = 8). Prior to co-culture, Der.p1-induced mo-DCs were stimulated with IL-25, IL-33, and TSLP, singly or in combination, for 24 h. **(B)** Quantification of percentages of IFN-γ^+^ and IL-17^+^ cells in CD4^+^ T cells. Data are expressed as means ± SD. Significant differences were tested using the paired *t*-test between control and cytokine stimulation groups, and using Student’s *t*-test between cytokine stimulation groups.

We next focused on the effects of the cytokine triad on the function of AR-derived mo-DCs induced by Der.p1. The mo-DCs were stimulated with IL-25, IL-33, and TSLP, alone or in combination, for 24 h, and then co-cultured with CD4^+^ T cells sorted from HC subjects for 5 days. No significant change in CD69 expression on the sorted CD4^+^ T cells was observed compared with their counterparts in PBMCs (Figure 4). We found that IL-25, IL-33, and TSLP, alone or in double combination, significantly increased the production of Th2 cytokines including IL-4, IL-5, and IL-13, both intracellularly, as evaluated by flow cytometry (Figures 5A,B), and extracellularly, as measured by ELISA (Figure 5C). IL-4, IL-5, and IL-13 responses were further enhanced when mo-DCs were stimulated with a triple combination of IL-25, IL-33, and TSLP (Figures 5A–C). However, we observed no significant change in IFN-γ or IL-17A production in CD4^+^ T cells between mo-DCs treated with or without IL-25, IL-33, and TLSP, alone or in combination (Figure 6).

**FIGURE 7:**
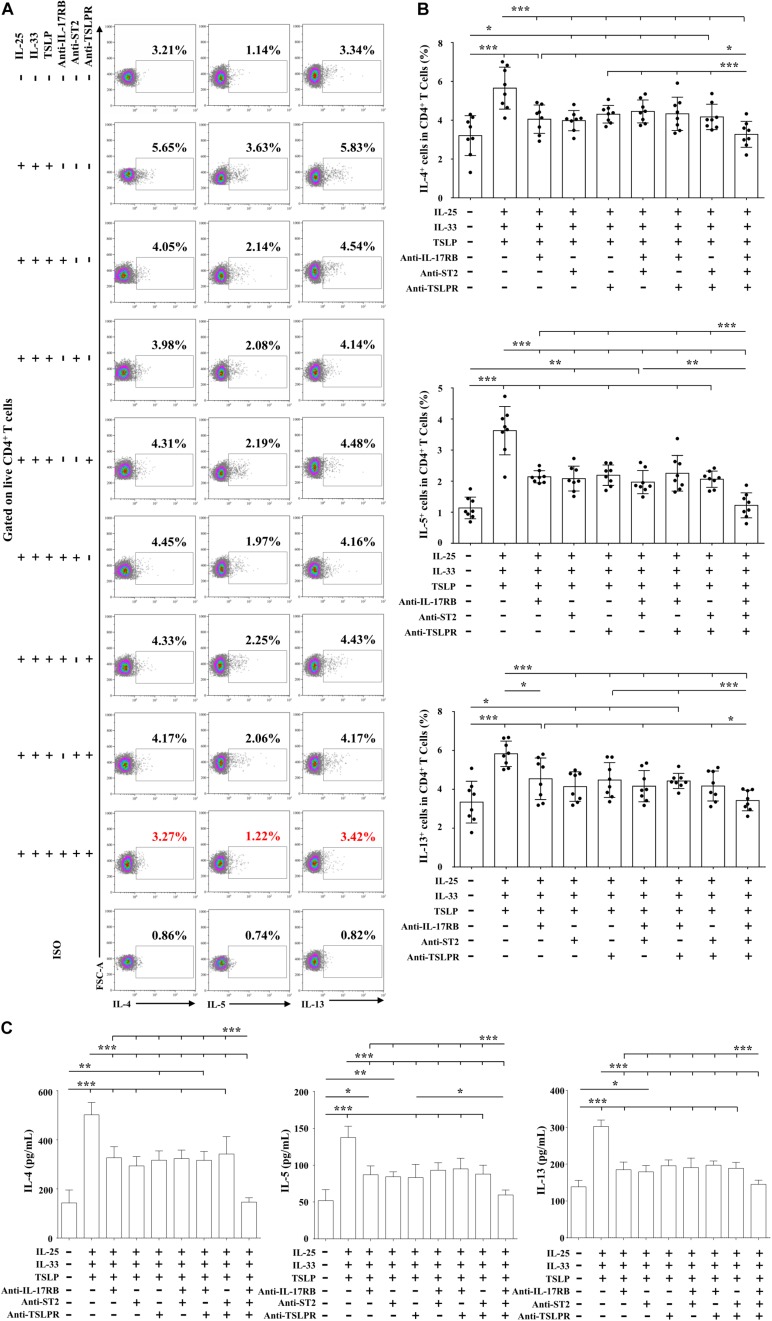
Combined pro-Th2 effects of IL-25, IL-33, and TSLP on AR-derived mo-DCs. **(A)** Representative flow plots of IL-4, IL-5, and IL-13 expression by CD4^+^ T cells co-cultured for 5 days with Der.p1-induced mo-DCs from AR subjects (*n* = 8). Prior to co-culture, mo-DCs were induced by Der.p1 in the presence of anti-IL-17RB, anti-ST2, and anti-TSLPR blocking antibodies, singly or in combination, for 48 h, and then further stimulated with combined IL-25, IL-33, and TSLP for 24 h. **(B)** Quantification of percentages of IL-4^+^, IL-5^+^, and IL-13^+^ cells in CD4^+^ T cells. **(C)** Protein levels of IL-4, IL-5, and IL-13 in the supernatants of co-cultured cells, as measured by ELISA (*n* = 6). Data are expressed as means ± SD. Significant differences were tested using the paired *t*-test between control and antibody blocking groups, and using Student’s *t*-test between antibody blocking groups. *P < 0.05, **P < 0.01, ***P < 0.001.

To further confirm the combined pro-Th2 effect of IL-25, IL-33, and TSLP, we used anti-IL-17RB, anti-ST2, and anti-TSLPR on day 5, alone or in combination, together with Der.p1 stimulation, to block IL-25, IL-33, and TSLP signals. As expected, when signals from the triad of cytokines were blocked singly or in double combination, IL-4, IL-5, and IL-13 responses were impaired (Figures 7A–C). However, the decreases in Th2 cytokine level were similar between those blocked singly and in double combination (Figures 7A–C). Only when the triad of cytokine signals was blocked in triple combination were IL-4, IL-5, and IL-13 responses suppressed to negative control levels (Figures 7A–C).

### IL-17RB, ST2, and TSLPR Upregulation in Myeloid DCs Was Associated With AR Severity

**FIGURE 8:**
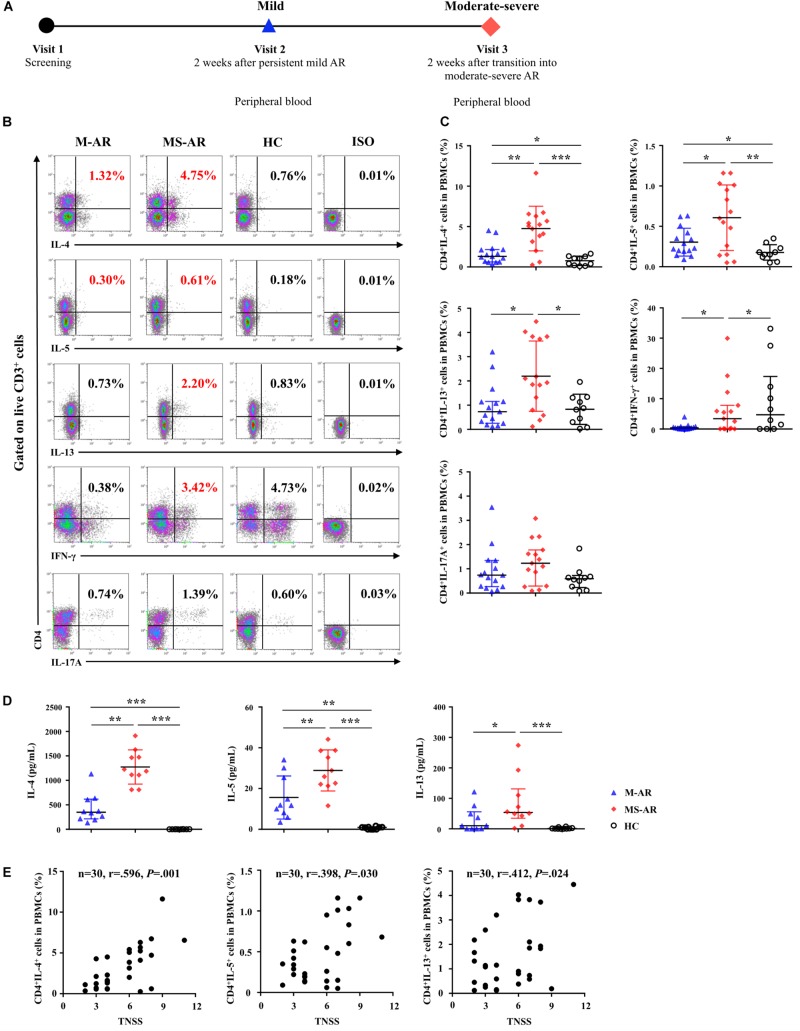
Enhanced Th2 response, but not IL-25, IL-33, and TSLP levels, was associated with disease exacerbation in AR patients. **(A)** Flow diagram for follow-up study of AR patients. **(B)** Representative flow plots of IL-4, IL-5, IL-13, IFN-γ, and IL-17 expression by CD4^+^ T cells in PBMCs from AR subjects in both mild phase (M-AR) (*n* = 15) and moderate to severe phase (MS-AR) (*n* = 15), and HC (*n* = 10) subjects. **(C)** Quantification of percentages of IL-4^+^, IL-5^+^, IL-13^+^, IFN-γ^+^, and IL-17^+^ cells in CD4^+^ T cells in PBMCs. **(D)** Protein levels of IL-4, IL-5, and IL-13 in serum of AR (*n* = 15) and HC (*n* = 10) subjects, as measured by ELISA. Data were tested for normality using the Shapiro–Wilk test, and expressed as means ± SD or medians with interquartile range (IQR), according to normality test results. Significant differences were tested using Student’s *t*-test or the Mann–Whitney *U* test between HC and AR groups, and using the paired *t*-test or Wilcoxon matched pairs signed rank test between M-AR and MS-AR groups. **(E)** Correlation of Total Nasal Symptom Score (TNSS) with percentage of IL-4^+^, IL-5^+^, and IL-13^+^ T cells in PBMCs among M-AR and MS-AR patients (*n* = 30) were analyzed using Spearman’s rank correlation coefficient (*r* = 0.596, *P* = 0.001, 1–β = 0.952; *r* = 0.398, *P* = 0.030, 1–β = 0.604; and *r* = 0.412, *P* = 0.024, 1–β = 0.638, respectively). *P < 0.05, **P < 0.01, ***P < 0.001.

Since Der.p1 was shown to best induce IL-17RB, ST2, and TSLPR expression on AR-derived mo-DCs (Figure 1), we next explored whether DC expression of these receptors was associated with disease severity in patients with HDM-sensitive AR. M-AR patients were enrolled and followed up for disease exacerbation (Figure 8A). As expected, IL-4, IL-5, and IL-13 responses were elevated in patients with AR, as measured by intracellular flow cytometry and ELISA (Figures 8B–D). Despite worsening of patient symptoms from M-AR to MS-AR, IL-4, IL-5, and IL-13 responses were further enhanced (Figures 8B–D). Correlation analysis showed that percentages of IL-4^+^, IL-5^+^, and IL-13^+^ T cells were positively correlated with TNSS in AR patients (*r* = 0.596, *P* = 0.001; *r* = 0.398, *P* = 0.030; and *r* = 0.412, *P* = 0.024, respectively) (Figure 8E). We observed no significant difference in IFN-γ and IL-17A responses, as measured by intracellular flow cytometry between AR and HC subjects. Interestingly, we observed elevated IFN-γ production in CD4^+^ T cells among MS-AR patients compared with M-AR patients (Figures 8B,C).

**FIGURE 9:**
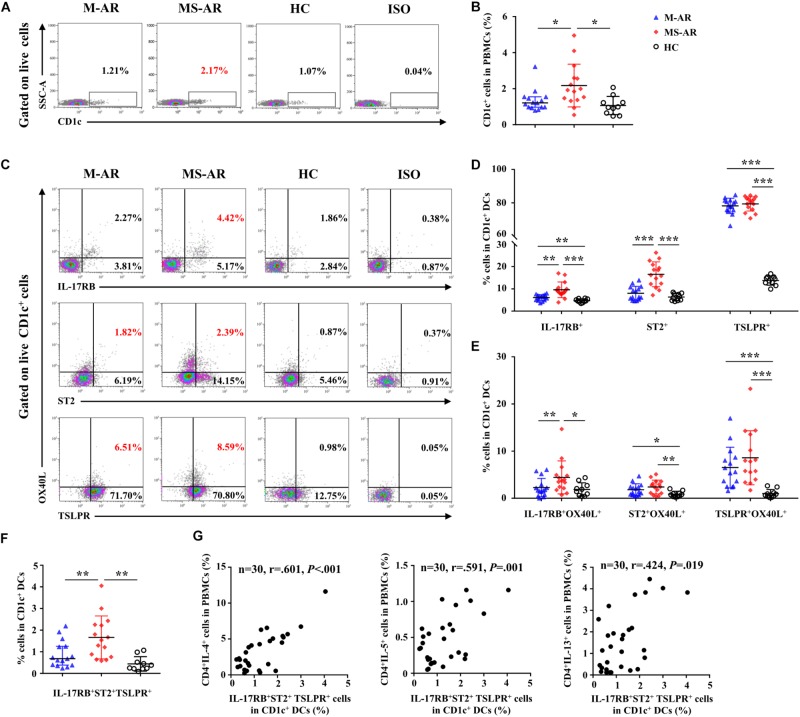
Upregulation of IL-17RB, ST2, and TSLPR on myeloid DCs (mDCs) was associated with AR disease severity. **(A)** Representative flow plots of the percentage of CD1c^+^ cells in PBMCs from M-AR (*n* = 15), MS-AR (*n* = 15), and HC (*n* = 10) subjects. **(B)** Quantification of the percentage of CD1c^+^ cells in PBMCs. **(C)** Representative flow plots of the percentage of IL-17RB^+^, ST2^+^, TSLPR^+^, and OX40L^+^ cells in CD1c^+^ mDCs of PBMCs from M-AR (*n* = 15), MS-AR (*n* = 15), and HC (*n* = 10) subjects. **(D)** Quantification of percentages of IL-17RB^+^, ST2^+^, and TSLPR^+^ cells in CD1c^+^ mDCs of PBMCs. **(E)** Quantification of OX40L expression in IL-17RB^+^, ST2^+^, and TSLPR^+^ mDCs in PBMCs. **(F)** Quantification of the percentage of IL-17RB^+^ST2^+^TSLPR^+^ cells in CD1c^+^ mDCs of PBMCs. Data were tested for normality using the Shapiro–Wilk test, and expressed as means ± SD or medians with IQR, according to normality test results. Significant differences were tested using Student’s *t*-test or the Mann–Whitney *U-*test between HC and AR groups, and using the paired *t*-test or Wilcoxon matched pairs signed rank test between M-AR and MS-AR groups. **(G)** Correlation of the percentage of IL-17RB^+^ST2^+^TSLPR^+^ mDCs with that of IL-4^+^, IL-5^+^, and IL-13^+^ T cells in PBMCs of M-AR and MS-AR patients (*n* = 30) were analyzed using Spearman’s rank correlation coefficient (*r* = 0.601, *P* < 0.001, 1–β = 0.956; *r* = 0.591, *P* = 0.001, 1–β = 0.948; and *r* = 0.424, *P* = 0.019, 1–β = 0.667, respectively). *P < 0.05, **P < 0.01, ***P < 0.001.

We next detected IL-17RB, ST2, and TSLPR expression levels on CD1c^+^ mDCs in peripheral blood. As shown by flow cytometry, the percentage of mDCs in PBMCs was elevated in MS-AR patients, but not in M-AR patients, compared with HC subjects (Figures 9A,B). IL-17RB levels on mDCs were increased in both M-AR and MS-AR patients (Figures 9C,D). IL-17RB levels on mDCs of MS-AR patients were significantly higher than those of M-AR patients (Figures 9C,D). ST2 levels on mDCs were increased in MS-AR patients, but not in M-AR patients, compared with HC subjects. The percentage of TSLPR^+^ cells in mDCs was as high as 80% in both M-AR and MS-AR patients, which was significantly higher than that of HC subjects (Figures 9C,D). IL-17RB^+^ mDCs in MS-AR patients, but not M-AR patients, expressed higher OX40L levels than HC subjects (Figure 9E). In addition, both ST2^+^ mDCs and TSLPR^+^ mDCs in M-AR and MS-AR patients expressed higher OX40L levels than HC subjects (Figure 9E).

We further analyzed IL-17RB, ST2, and TLSPR co-expression on mDCs and explored their correlation with the Th2 response. The results showed that the percentage of mDCs co-expressing IL-17RB, ST2, and TSLPR was significantly increased in both M-AR and MS-AR patients compared with that of HC subjects (Figure 9F). The percentage of mDCs co-expressing IL-17RB, ST2, and TSLPR was significantly higher in MS-AR patients than in M-AR patients (Figure 9F). Correlation analysis showed that the percentage of IL-17RB^+^ST2^+^TSLPR^+^ mDCs was positively correlated with that of IL-4^+^, IL-5^+^, and IL-13^+^ T cells in AR patients (*r* = 0.601, *P* < 0.001; *r* = 0.591, *P* = 0.001; and *r* = 0.424, *P* = 0.019, respectively) (Figure 9G).

## Discussion

TSLP, IL-25, and IL-33 are three epithelial-derived innate cytokines that have been independently reported to be activators of type 2 immunity (Fort et al., 2001; Soumelis et al., 2002; Schmitz et al., 2005; Cheng et al., 2014; Christianson et al., 2015; Hong et al., 2018). However, their potency as standalone stimulators of DCs or their potential mutual redundancy remains unclear. Since DCs closely underlie the mucosa epithelium (Hammad and Lambrecht, 2011), a better understanding of the potency of this triad of epithelial-derived cytokines as DC activators could be critical when selecting the most efficacious therapeutic targets for atopic diseases such as AR and asthma.

Recently, we demonstrated that IL-17RB^+^ mo-DCs derived from AR patients can be induced by LPS, and displayed enhanced pro-Th2 function following IL-25 stimulation (Zheng et al., 2017). In the current study, we used the same methodology to rapidly generate immature DCs from peripheral blood monocytes, and found that nine human TLR1–9 ligands were all able to upregulate IL-17RB, ST2, and TSLPR by varying degrees on immature mo-DCs generated from AR patients sensitized to HDMs. Der.p1 showed the strongest induction of IL-17RB, ST2, and TSLPR. Using AR-derived mo-DCs, we found that, although individual activation of IL-17RB, ST2, and TSLPR potentiated the Th2-polarizing function of AR-derived mo-DCs, triple combinatorial activation was more efficacious than activating any of these receptors alone or in double combination. Interestingly, we detected no significant difference when the receptors were activated alone or in double combination. IL-17RB, ST2, and TSLPR levels were upregulated in mDCs in AR patients and associated with disease severity. Together, these data reveal that the pro-Th2 function of DCs derived from AR patients by combinatorial activation of IL-17RB, ST2, and TSLPR signaling is superior to that by single or double activation, suggesting that aggressive triple targeting of these receptors may more effectively inhibit DC-driven type 2 inflammation in patients with AR.

It has been reported that IL-17RB, ST2, and TSLPR can be expressed on a variety of immune cells such as innate lymphoid cells (Hazenberg and Spits, 2014; Stier and Peebles, 2017), T cells (Omori and Ziegler, 2007; Rochman et al., 2007; Lam et al., 2016), and DCs (Ito et al., 2005; Turnquist et al., 2008; Rank et al., 2009; Zheng et al., 2017). DC expression of these receptors has been described in our previous work and by others as being related to DC-driven type 2 immune responses (Ito et al., 2005; Turnquist et al., 2008; Rank et al., 2009; Zheng et al., 2017). However, little is known about the regulation and function of DC expression of these receptors in allergic diseases. In the present study, we showed that mo-DCs derived from AR patients, but not HCs, upregulated IL-17RB, ST2, and TSLPR expression in response to TLR ligands, including those for TLR1–9. In addition, OX40L, a costimulatory molecule of the TNF ligand family, which has been described on the surfaces of DCs as supporting a Th2-skewed immune response (Ito et al., 2005; Jenkins et al., 2007), was increased only on AR-derived mo-DCs in response to Der.p1 stimulation. We confirmed *in vivo* that CD1c^+^ DCs in PBMCs of AR patients expressed higher IL-17RB, ST2, and TSLPR levels than their HC counterparts, which is consistent with our previous report (Zheng et al., 2017). Levels of CD1c^+^ DCs coexpressing IL-17RB, ST2, and TSLPR were positively correlated with those of Th2 cells in PBMCs. Together, these results suggest that DC expression of IL-17RB, ST2, and TSLPR may be involved in the regulation of Th2 inflammation in AR patients.

Although previous studies have identified critical roles for TSLP, IL-25, and IL-33 as important initiators of the type 2 response or inflammation, few studies have addressed their overlapping effects, particularly on the T cell-polarizing function of DCs. In mouse models of allergen-induced airway inflammation and acute pulmonary fibrosis, individual blockade of IL-25, IL-33, or TSLP was shown to have little influence on Th2-mediated pulmonary fibrosis, whereas blockade of all three cytokines significantly reduced inflammation, ILC2 recruitment, eosinophilia, and fibrosis in the lung, suggesting that combined targeting may be required to temper type 2-driven inflammation (Vannella et al., 2016). In another study using a food allergy mouse model, blockade of IL-25, IL-33, or TSLP did not suppress established food allergies, and optimal food allergy suppression required treatment by combined blocking of all three cytokine signals (Khodoun et al., 2018). In the current study, we found that collective activation of IL-17RB, ST2, and TSLPR on AR-derived mo-DCs resulted in a stronger Th2 response than their single or double-combination activation, suggesting that successful inhibition of cytokine triad-elicited Th2 inflammation would require inhibition of all three cytokine signals or suppression of the production of all three cytokines. This hypothesis was further supported by the results of an *in vitro* blocking experiment showing that inhibition of the Th2 response elicited by collective activation of IL-17RB, ST2, and TSLPR on AR-derived mo-DCs required the blocking of all three signals.

Although we have provided new information that combinatorial activation of IL-17RB, ST2, and TSLPR on DCs results in more potent promotion of the Th2 response, several limitations must be addressed before more definitive conclusions can be drawn. The major limitation of this finding is that it lacks an *ex vivo* study. This limitation may be addressed in future work through DC adoptive transfer experiments. In addition, because the DCs used in the present study were induced from peripheral blood monocytes, which are unable to represent distinct DC subsets such as myeloid or plasmoid DCs, the roles of IL-17RB, ST2, and TSLPR in the function of different DC subsets remain to be investigated.

In summary, we demonstrated that AR-derived mo-DCs activated by triple combination of IL-17RB, ST2, and TSLPR have a stronger capacity to elicit the Th2 response than those activated singly or in double combination. Additionally, mDCs in PBMCs of AR patients express higher levels of IL-17RB, ST2, and TSLPR, in a manner positively correlated with disease severity and Th2 cell levels in peripheral blood, suggesting that the Th2 response amplification function of DCs by combinatorial IL-17RB, ST2, and TSLPR signaling is associated with AR severity.

## Data Availability Statement

The datasets generated for this study are available on request to the corresponding author.

## Ethics Statement

The studies involving human participants were reviewed and approved by the Ethics Committee of the First Affiliated Hospital, Sun Yat-sen University. All patients provided their written informed consent before participation in this study.

## Author Contributions

RZ, YC, and JS performed volunteer screening, cell culture, flow cytometry, ELISA, and data analysis and prepared the manuscript. KW and XH participated in data analysis and manuscript preparation. YS and QY designed the study and prepared the manuscript. All authors reviewed and approved the final manuscript.

## Conflict of Interest

The authors declare that the research was conducted in the absence of any commercial or financial relationships that could be construed as a potential conflict of interest.
